# Supplementary material for: Dynamics of Cyclooxygenase-1 Positive Microglia/Macrophage in the Retina of Pathological Model Mice as a Biomarker of the Retinal Inflammatory Diseases
Source: Int J Mol Sci. 2021 Mar 25;22(7):3396. doi: 10.3390/ijms22073396 (PMC8036698; doi:10.3390/ijms22073396)
Supplement: Supplementary file 1 [file ijms-22-03396-s001.pdf]

International Journal of Molecular Sciences

Supplemental Information

Dynamics of cyclooxygenase-1 positive microglia/macrophage in the retina of pathological model mice as a biomarker of the retinal inflammatory diseases

Kenichi Makabe, Sunao Sugita, Yoko Futatsugi, and Masayo Takahashi

Inventory of Supplemental Information

Figure S1, resting microglia in normal retina
Figure S2, microglia/macrophage activation in NMDA retinal neurotoxicity model
Figure S3, microglia/macrophage activation in LPS treated ocular inflammation model
Figure S4, microglia/macrophage activation in EAU model

Supplemental Figure 1

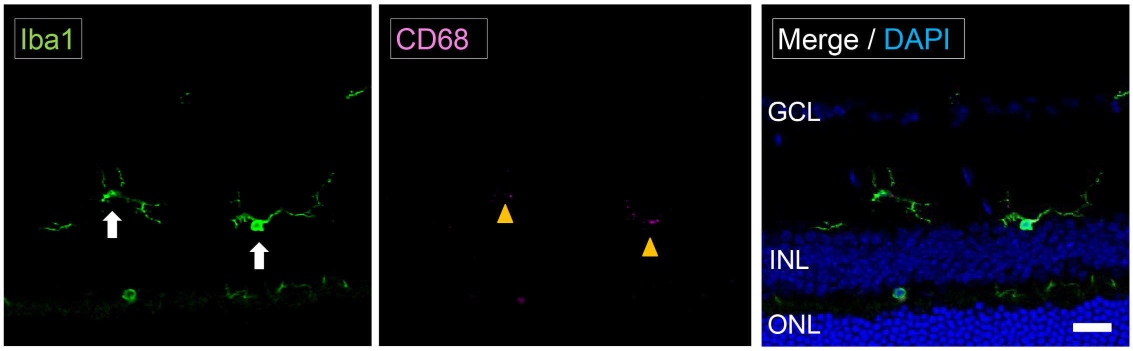

Figure S1. Microglia at resting state in the retina of normal mouse.
Photomicrographs of the retina in normal mouse labeled with Iba1 (microglia/macrophage marker; left) and CD68 (activated microglia/macrophage marker; middle). The Iba1^{+} cells were ramified microglia with long processes (white arrows). Immunopositivity of CD68 were very weak (yellow arrowhead).
Scale bar, 20μm.

Supplemental Figure 2

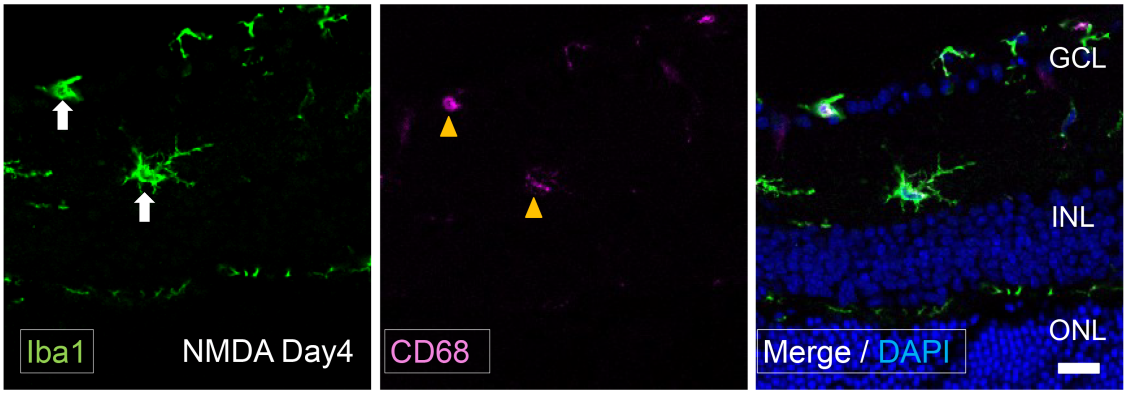

Figure S2. Active microglia/macrophage in the retina of NMDA retinal neurotoxicity model.

Photomicrographs of the retina in NMDA retinal neurotoxicity model mice 4 days after NMDA-administration (NMDA Day 4) labeled with Iba1 (left) and CD68 (middle).
The processes of Iba1^{+} cells were shorter and thicker than normal (white arrows), and the cells were CD68 positive (yellow arrowheads).
Scale bar, 20μm.

Supplemental Figure 3

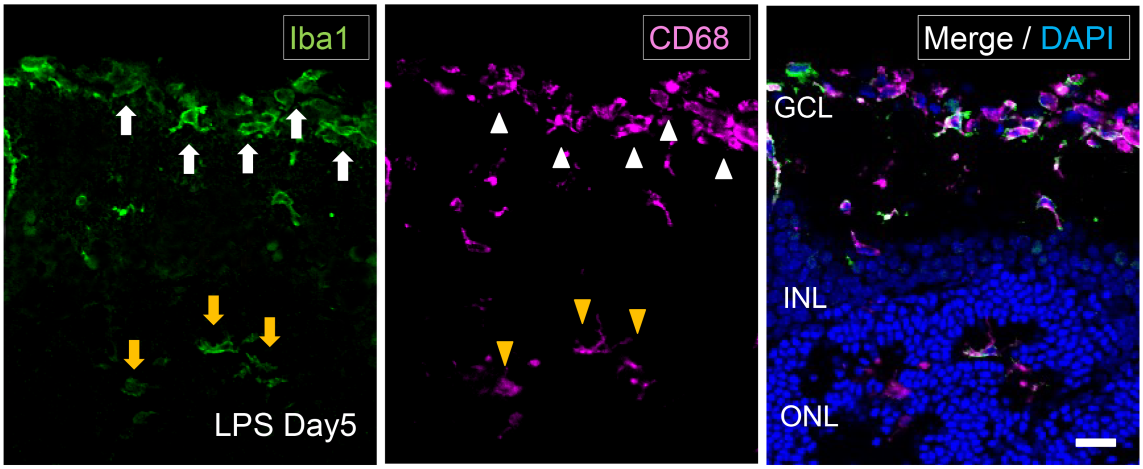

Figure S3. Activation and migration of microglia/macrophage in the retina of LPS treated ocular inflammation model.

Photomicrographs of the retina in LPS treated ocular inflammation model mice 5 days after LPS-administration (LPS Day 5) labeled with Iba1 (left) and CD68 (middle).
Iba1-positive amoeboid cells accumulated around the GCL (white arrows), and were CD68 positive (white arrowheads). There were Iba1-positive amoeboid cells that infiltrated into the ONL (yellow arrows), which were also CD68 positive (yellow arrowheads). Scale bar, 20μm.

Supplemental Figure 4

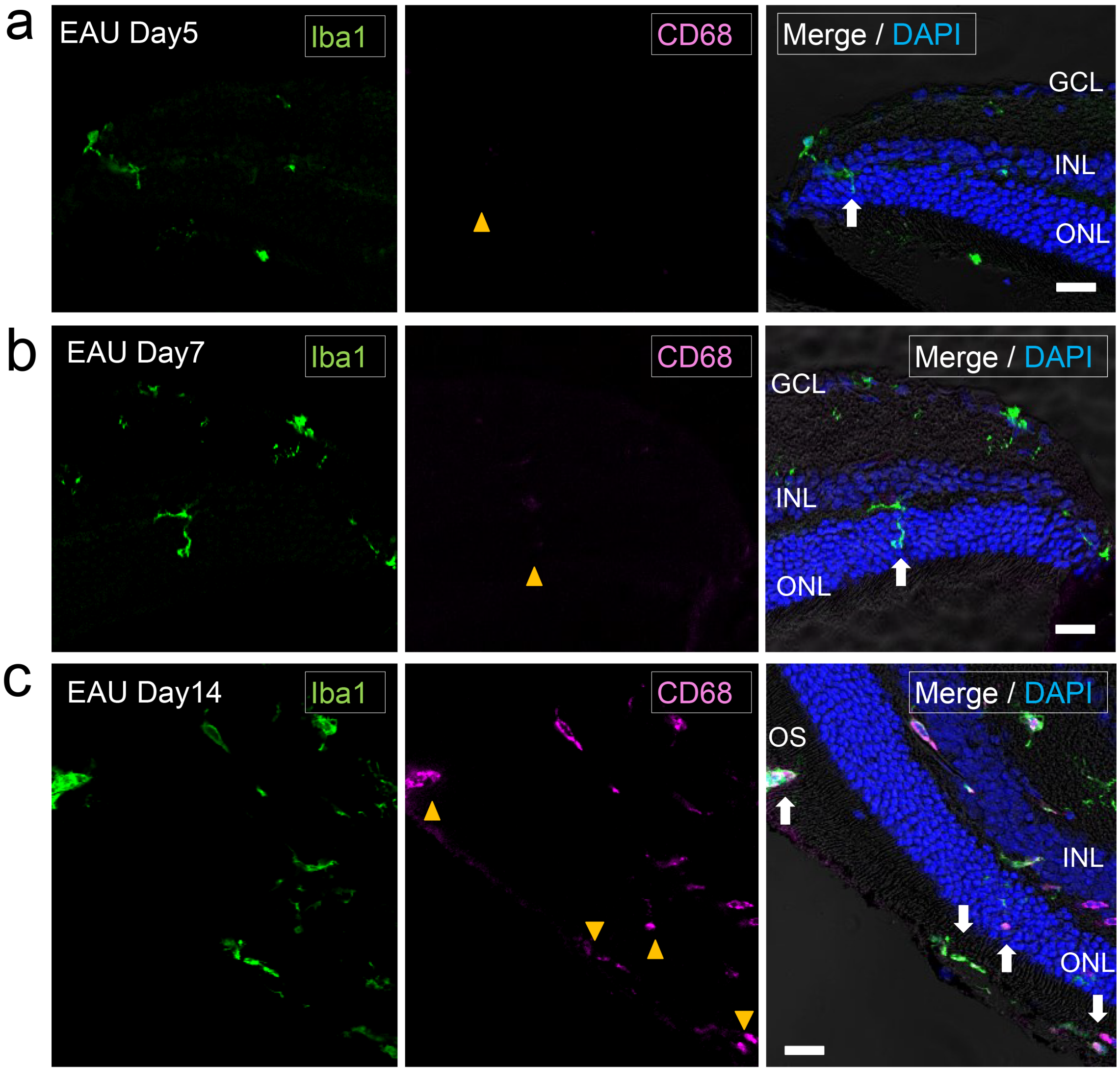

Figure S4 Activation and migration of microglia/macrophage in retina of EAU model
Photomicrographs of the retina in EAU model mice labeled with Iba1 (left) and CD68 (middle).

(a) Five days after immunization (Day 5), Iba1^{+} lamified cells with processes had infiltrated into the ONL (white arrow). These cells were CD68 negative (yellow arrowhead). Scale bar, 20μm.

(b) On Day 7, Iba1$^+$ lamified cells with processes had infiltrated into the ONL (white arrow). CD68 was negative in them (yellow arrowhead). Scale bar, 20μm.
(c) On Day 14, Iba1$^+$ amoeboid cells infiltrating into the ONL and the OS were observed (white arrows). These cells were CD68 positive (yellow arrowheads). Scale bar, 20μm.
